# Comparison between two asynchronous teaching methods in an undergraduate dental course: a pilot study

**DOI:** 10.1186/s12909-022-03557-7

**Published:** 2022-06-23

**Authors:** Fahad Alharbi, Saleh H. Alwadei, Abdurahman Alwadei, Saeed Asiri, Farhan Alwadei, Ali Alqerban, Mohammed Almuzian

**Affiliations:** 1 Department of preventive dental sciences/College of Dentistry, Prince Sattam Bin Abdulaziz University, Al-Kharj, 11942 Saudi Arabia; 2 Department of Pediatric Dentistry and Orthodontics, College of Dentistry, King Saud University, Riyadh, Saudi Arabia; 3 Private clinic, Edinburgh, UK

**Keywords:** Learning, Dental education, Teaching methods, Dental students

## Abstract

**Background:**

Properly designed and implemented eLearning can lead to improvement of dental teaching quality. Various strategies have been proposed to increase the effectiveness of eLearning in dental education, however, there is a lack of research to assess the effectiveness of these strategies.

**Objective:**

To investigate dental students’ learning performance and perception of a virtual flipped learning format compared to a virtual traditional learning method.

**Methods:**

A crossover pilot study was conducted at the College of Dentistry, Princes Sattam Bin Abdulaziz University, Saudi Arabia. Computer-generated randomization, blinded from researchers who analyzed the results, was performed to allocate 32 participants (aged 23.27 ± 0.86 years) to one of two groups. Participants in the control group were taught through the virtual traditional learning method (VTL) using live video lectures. In contrast, participants in the intervention group were taught through the virtual flipped learning method (VFL) using recorded online lectures and post-lecture virtual discussions. Learning gain and preference were measured by pre- and post-test average score differences and a modified validated survey, respectively.

**Results:**

There was no significant difference in learning performance between VFL and VTL groups (*P* > 0.05). However, students preferred VFL over VTL and the differences were significant among all survey items, except for the opportunity to ask questions.

**Conclusion:**

Health professions educators are encouraged to carefully design online curricula with efficient learning strategies that help students improve learning performance and foster self-directed learning skills while valuing active learning in an online environment.

**Trial registration:**

NCT04692142, 31/12/2020.

**Supplementary Information:**

The online version contains supplementary material available at 10.1186/s12909-022-03557-7.

## Background

Higher education has been greatly influenced by globalization, technological advances, competition for eLearning opportunities and reduced public funding [1]. Recently, eLearning has been mainstreamed in health profession education, including dental, medical, nursing, and other allied healthcare education [2]. eLearning encompasses three models: 1) adjunct model where face-to-face learning is supported by online learning, 2) blended learning where face-to-face learning is integrated with online learning, and 3) pure online model where all learning content is provided via technology without face-to-face learning, providing greatest independence to learners [3, 4]. Individual and/or collaborative learning can take place within the pure online model, with collaborative learning being delivered synchronously (virtual or face-to-face) or asynchronously (text-based internet) [3, 4]. Researchers have proposed various components of eLearning based on different factors, including content delivery (asynchronous vs. synchronous), mode of delivery (online-only vs. blended), and learner independence (individual vs. collaborative) [5]. According to Omar et al. (2011), asynchronous eLearning is the delivery of learning content at different times than receipt by learners, while synchronous eLearning entails simultaneous content delivery as receipt by learners. With regards to independence, individual versus collaborative learning means that learners work either independently or collaboratively with one another to complete learning tasks [6].

Several randomized controlled trials examined the effects of eLearning versus traditional learning. Higher satisfaction with eLearning with little or no differences regarding knowledge and learning experiences was reported [7–9]. However, due to the asynchronous nature of eLearning where in-person interactions are reduced, students reported a lack of confidence in understanding the material [10, 11]. Such lack of engagement presents a pedogeological challenge that can be addressed with blended learning or flipped classroom models, the benefits of which stem mainly from the active in-class learning component [12]. In the conventional flipped learning (FL) model, face-to-face complementary instruction is scheduled sequentially to what students learn independently. Thus, in-class time is typically designed to focus on aspects of the content for which students encountered difficulty or misconceptions.

Amid the COVID 19 pandemic, embracing educational technology became necessary [13, 14]. In addition to the breadth and depth of a university curricular content, the pandemic-led reality check heightened a long-overdue realization by academic leaders and teachers on the importance of eLearning and the need to adopt it as a scaled-up educational tool to facilitate the dissemination of a pedagogy-driven curriculum across time and space [15–17]. Recently published literature has demonstrated how the FL model can be modified and adapted to meet the virtual model [14, 16, 18, 19], herein referred to as virtual flipped learning (VFL). In the VFL model, the teacher provides didactic material as a prerecorded video lecture for students to watch at any time prior to a virtual learning session [18]. Similar to the in-class component of the conventional flipped classroom model, virtual learning sessions focus primarily on synthesis and application, as well as clarification of concepts that are difficult to grasp independently [18]. Such modification has allowed educators to eliminate the “classroom” while maintaining an appropriate level of interaction among learners through Internet communication tools and platforms [18].

The phrase “passive learning” describes the traditional approach to teaching and learning, in which the teacher lectures while the learner passively receives information [20]. On the other hand, active learning entails information being generated by the learners themselves who are engaged during the learning process through small group work and collaboration with other learners and the instructor [21]. Others have emphasized that active learners deliberately decide about what they learn [22]. The flipped classroom model (either conventional or virtual) asks learners to shift the knowledge acquisition and application of learning, which has the potential to address key academic challenges in the context of health profession education. These challenges include dissemination of a flexible and adaptable curriculum, minimizing information overload, fostering learner preferences [14, 15, 20, 22], and perhaps most significantly, maintaining and facilitating active learning in the digital era of learning, especially due to the demands inflicted by the COVID-19 pandemic [20]. Proponents of this view suggest that there is a greater focus on self-learning the lecture or theory material during the pre-class (at-home) activities of learning as well as active collaborative learning practices during the in-class time (or virtual learning sessions), which often involve group learning, discussions, interactive exercises, and case studies [22, 23]. Table 1 highlights the differences across the various learning models mentioned above with regard to knowledge acquisition and the application of learning.

**Table 1:** Knowledge acquisition and application stages of learning across different learning models

Learning Model	Knowledge acquisition	Application and practice
**Conventional passive learning**	Face-to-face (lecture)	Self-directed (homework)
**Conventional active learning**	Face-to-face (Discussions, interactive exercises, case studies)	Collaborative (groupwork)
**Conventional online learning**	Digital – synchronous or asynchronous (Live or pre-recorded lecture)	Self-directed (homework)
**Conventional flipped learning**	Self-directed (prework)	Face-to-face (Discussions, interactive exercises, case studies)
**Virtual flipped learning**	Self-directed (prework/pre-recorded lecture)	Virtual (Discussions, interactive exercises, case studies)

Modified from J. Phillips and F. Wiesbauer (2022) [20]

Although the benefits of active learning over passive face-to-face learning have been examined and established in education literature [24], it is still ambiguous if this difference prevails in an online learning environment [25]. Moreover, the implementation of active learning practices and collaboration can be perceived as challenging in an online learning environment [21]. Little empirical evidence exists on what entails an efficient delivery of online learning material based on sound pedagogical frameworks, standardized learning principles, and learning theories and designs [2]. Others have highlighted two arguments concerning the FL method, including; 1) time and commitment needed to create a meaningful educational experience and the contextual variability associated with such experiences, and 2) lack of evidence with regard to knowledge retention compared with traditional classrooms [20]. Both limitations are especially applicable to a novel approach such as the VFL method [18].

Currently, the unprecedented circumstance of the pandemic has shifted the way instructors think about teaching and learning, which may ultimately last in the foreseeable future. One thing is certain; that is, VFL does not equate to conventional flipped learning. VFL is not merely integrating technology or facilitating learning access but is fundamentally about recognizing and reforming the relationship between teaching and learning as it occurs in a digital environment. The conceptual benefits of VFL to achieve efficient and convenient learning experiences are apparent. However, in the context of dental education, and to the best of the authors’ knowledge, there is no empirical research available on the effectiveness of the VFL method with regards to learning outcomes and student satisfaction. Rigorous research is needed to explore the changes being made by instructors and educators during the pandemic, which may produce hypotheses about the most efficient methods for dental education and potentially be implemented in the future to guide innovative solutions in dental education. Therefore, this pilot study establishes a proof of concept by hypothesizing that the VFL method is at least as effective as passive online learning reflected by live video lectures only, herein referred to as the virtual traditional learning (VTL) method. To address this aim, we sought to examine the VFL method in an orthodontic course and study its impact on students’ short-term recall knowledge of the lectures’ content and the perceived value of their learning experience in comparison to the VTL method.

## Methods

### Design

The trial was designed as a crossover clustered randomized trial whereby each group/ cluster acted as their control for their learning performance and perception.

### Setting

College of Dentistry, Princes Sattam Bin Abdulaziz University, Alkharj, Saudi Arabia.

### Consents

The study was carried out in accordance with the ethical guidelines for scientific research of Prince Sattam Bin Abdulaziz University. The ethical approval was granted from the Scientific Research Unit at the College of Dentistry, Prince Sattam Bin Abdulaziz University, Alkharj (ethical approval: PSAU2020030) after a full review. A presentation explaining the study by one of the researchers (FA) was given to the students before deciding to participate in the study. The researcher (FA) then asked the participants to sign a written informed consent form. The informed consent from was signed by all participants before the commencement of the study. The study was registered with ClinicalTrial.gov (Identifier: NCT04692142, 31/12/2020).

### Participants

Fourth-year undergraduate dental students enrolled in the preclinical orthodontic course at Prince Sattam University were invited to participate in the study. Students who were registered in the course for the second time were excluded to lessen the bias associated with their previous experiences. The sample size was based on the total number of students registered in the course at the time of the study.

### Randomization

Computer-generated randomization was performed to allocate participants to one of two groups. The sequence of random allocation was concealed from researchers who analyzed the results. Participants in the control group were taught through the VTL method using live-video streaming, while participants in the intervention group were taught through the VFL method. The VTL group included 16 students, while the VFL group included 17 students. We use the term control group to refer to students who studied using the VTL format method across both periods. We also use the term intervention group to refer to students who studied using the VFL format across both periods.

### Intervention

The intervention was implemented over three consecutive weeks. Four lectures were delivered asynchronously using two different teaching methods as mentioned above. Participants in VFL had monitored remote access to the recorded lectures for 1 week, followed by 4 additional small group online discussion sessions. The students in both groups remotely attended all pre-recorded and live-video virtual lectures.

### Intervention implementation rounds

#### Round I

The first lecture (L1: Adult Orthodontics) was taught in the first week. Before L1, both groups were asked to complete a set of multiple-choice questions (MCQs) formative exam (i.e., pre-test). Following the pre-test, participants allocated to the VFL arm were asked to leave the online platform. Then, the lecture was delivered to participants allocated to the VTL arm using a passive online teaching format (live-video streaming). L1 was also recorded after obtaining verbal consent from the attendees. After the lecture was completed, participants in the VTL group were asked to re-take the same pre-test exam and complete a questionnaire regarding the VTL experience.

Within 24 hours following L1, participants of VFL had 7-day monitored remote access to the recorded lecture of L1. At the expiry of remote access to the recorded lecture, participants from VFL were invited to attend 15-minute post-lecture discussion sessions about L1. To enhance the effectiveness of post-lecture discussions, participants in the VFL group were randomly divided into two smaller subgroups, with the same lecturer facilitating discussions within each group separately. At the end of each discussion session, participants were asked to re-take the pre-test of L1 and a complete questionnaire regarding their VFL experience.

#### Round II

Following the small group discussion sessions in round I, participants of VFL were given a 30-minute break before the start of the second lecture related to the topic of Orthognathic Surgery (L2). In this round (II), the intervention was crossed over. Hence, the cohort of VFL in round I acted as a control group while teaching L2 and received VTL intervention. On the other hand, the cohort who were taught using VTL in round I received VFL intervention. The same protocol of teaching models that were applied in round I was adopted in round II.

Participants of both groups were taught by the same instructor using the same materials, irrespective of the teaching methods. The lectures were delivered using the same presentation platform (PowerPoint, Microsoft Corp, Redmond, WA) and the same virtual learning platform (Zoom live-streaming software - Zoom Video Communications, Inc., San Jose, California, Version 5.4.3). The duration of each lecture was 60 minutes, while the post-lecture discussion in the VFL design lasted 15 minutes. Learning outcomes were based on each lecture’s learning objectives and outcomes that aligned with what is specified by the National Commission for Academic Accreditation and Assessment in Saudi Arabia.

### Study measures

Students’ performance was assessed by written pre- and post-tests of their short-term recall knowledge of the lectures’ content (periods 1 and 2). These tests included MCQs, which were administered electronically. The lecturer, who had taught the content for over 5 years, developed the tests with input from two coinvestigators (S.A and F.A) certified in item writing. The questions were tailored to their respective content to ensure specificity and were aligned with the item development guidelines provided by the Saudi Commission for Health Specialties. Learning effectiveness was measured by students’ performance on the post-test (positive-test raw scores). Learning gain was measured by the effect size of post- and pre-test average score differences between the VTL and the VFL groups. Using a modified validated survey, self-reported data from students provided valuable perspectives regarding students’ perspectives about their learning experiences with both instructional methods.

### Students’ perception

At the end of each lecture, students were asked to answer a questionnaire regarding their experience with the teaching methods they had received. We used a validated Web-Based Learning Environment Inventory (WELEI) questionnaire (Supplementary file 1), developed by Chang and Fisher [26]. In addition to the demographic section, the questionnaire consisted of items that captured the students’ perceptions of the web-based learning environments. Regarding the flipped classroom teaching method, we included additional questions adapted from previous studies that examined the students’ perceptions of a flipped classroom [27, 28]. The questionnaire was distributed electronically via SurveyMonkey (San Mateo, California, United States).

### Statistical analysis

The distribution of the raw data was investigated using the Shapiro-Wilk test of normality. The Wilcoxon signed-rank test was used to compare differences in median values between post- and pre-test scores for students under the VTL and VFL formats. In other words, we examined students’ learning improvement concerning different instructional modalities. The Mann–Whitney U test was used to compare differences in post-test score median values between the VTL and VFL groups. In other words, we examined the effectiveness of using VFL on students’ learning performance. The Cronbach’s alpha coefficient was used to assess the reliability of the questionnaires. Along with descriptive statistics to analyze students’ perception of their learning experiences, we conducted a Wilcoxon signed-rank test to compare differences in the students’ responses to individual/comparable items in the satisfaction/preference questionnaires between the VTL and VFL methods. Finally, we conducted correlation coefficient tests to examine the relationship between students’ overall satisfaction and preference with both instructional methods and their post-test scores. Data analysis was done with SPSS Statistics for Windows (version 27.0; IBM, Armonk, NY).

## Results

**Fig. 1:**
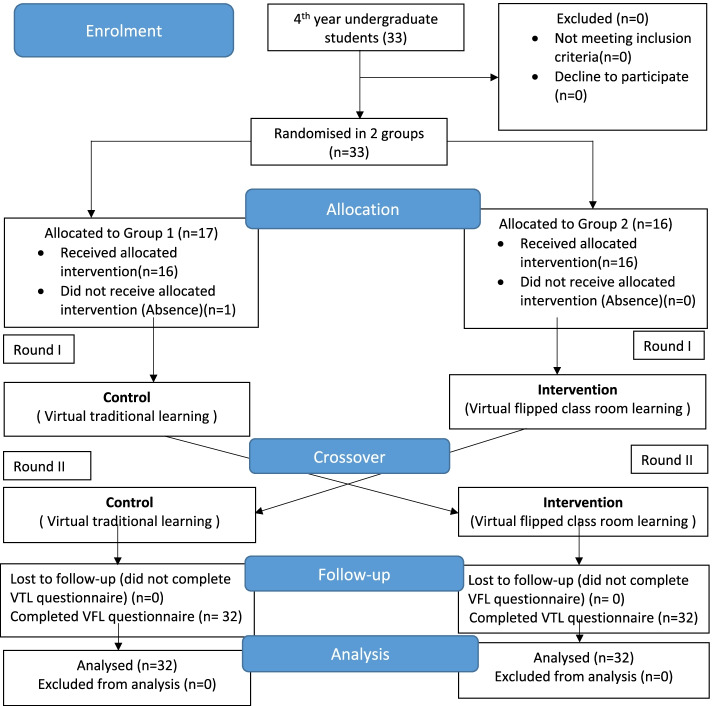
CONSORT flow diagram of the study

A total of 33 male dental students were enrolled in the preclinical orthodontic course at Prince Sattam University. Of these students, one student was absent at the time of the intervention. The final study sample was 32 students (23.27 ± 0.86 years). All students attended both lectures and completed the perception questionnaires (Fig. 1). The Shapiro-Wilk test of normality indicated the data was not normally distributed. The data also violated the assumptions for ANCOVA and one-way repeated measures ANOVA.

### Learning improvement within groups based on different instructional modalities

The Wilcoxon signed-rank test was used to identify student learning performance improvements after completing both lectures. Results showed significant improvement in post-test scores across the two instructional methods (*P* <  0.01). This indicates that, regardless of the instructional approach, both instructional methods effectively improved students’ short-term recall knowledge of the lectures’ content. The effect sizes for both groups under both instructional methods were great (*r* > 0.5). The effect size was slightly greater for group 1 under the VTL format (*r* = 0.61) compared with VFL (*r* = 0.59). The effect size for group 2 was similar for both instructional methods (*r* = 0.58). See Table 2.

**Table 2:** Improvement in learning performance within groups across both instructional modalities

Period (lecture)	N	Median	Paired Difference (post-test – pre-test)		
			z	r	***p***-value
**Period 1 – Control (VTL)**					
Pre-test	16	5.5	- 3.43	0.61	0.001
Post-test	16	9			
**Period 1 – Intervention (VFL)**					
Pre-test	16	5.5	- 3.08	0.59	0.002
Post-test	16	8			
**Period 2 – Control (VTL)**					
Pre-test	16	3.5	- 3.27	0.58	0.001
Post-test	16	9			
**Period 2 – Intervention (VFL)**					
Pre-test	16	4	- 3.32	0.58	0.001
Post-test	16	8.5			

### Differences in learning outcomes between the traditional online approach and the flipped online classroom approach

The Mann–Whitney U test was used to test the effectiveness of VFL on students’ learning performance, regardless of pre-test scores. For period 1, the results showed insignificant differences between the intervention group (Md = 8, *n* = 16) and the control group (Md = 5.5, *n* = 16, U = 93.5, z = − 1.33, *p* > 0.05). For period 2, the results also showed insignificant differences between the intervention group (Md = 8.5, *n* = 16) and the control group (Md = 9, *n* = 16, U = 122.5, z = − 0.22, *p* > 0.05). These findings indicate that the VFL format was not more effective than the VTL format, at least in relation to students’ performance on the post-test. Note that the median scores of the VTL format are greater than the VFL format for both periods. See Table 3.

**Table 3:** Differences in learning outcome between groups across both instructional modalities

Period (lecture)	N	Median	Sum of Ranks	Mann–Whitney U	z	***p***-value
**Period 1 – Control (VTL)**				93.5	− 1.326	.185
Pre-test Post-test	16	5.5	265			
	16	9	298.5			
**Period 1 – Intervention (VFL)**						
Pre-test	16	5.5	263			
Post-test	16	8	229			
**Period 2 – Control (VTL)**				122.5	−0.215	.829
Pre-test	16	3.5	243.5			
Post-test	16	9	269.5			
**Period 2 – Intervention (VFL)**						
Pre-test	16	4	284.5			
Post-test	16	8.5	258.5			

### Student perceptions of their learning experiences

The reliability of the Numerical Rating Scale questionnaires was measured using Cronbach’s alpha coefficient and indicated good internal consistency of items (Cronbach’s Alpha = 0.75). The Wilcoxon signed-rank test compared differences in the students’ responses to individual/comparable items in the perception questionnaires between the VTL and VFL approaches. Except for the opportunity to ask questions item (*p* > 0.05), there were statistically significant differences between the two instructional methods across all other comparable items (*p* > 0.01 and 0.05), with moderate-to-large effect sizes (*r* = 0.402 to 0.656). See Table 4. These findings indicate that students were generally more positive about their experience with the VFL format. Spearman’s rho correlation coefficients revealed insignificant correlations between students’ overall satisfaction and preference with both instructional methods and their post-test scores.

**Table 4:** Within individuals’ differences in perception of both instructional modalities

Item	N	Median		Paired Difference (VFL - VTL)		
		VTL	VFL	z	r	***p***-value
Do you feel that you understood the topic that was delivered today?	32	9.0	9.0	−2.481	0.439	0.013
How interesting did you find the seminar?	32	9.0	10	−3.173	0.561	0.002
Did you find it easy to concentrate?	32	7.0	9.0	−2.595	0.459	0.009
Was there an opportunity to ask questions?	32	10	10	−0.412	0.073	0.680
Did you find it easy to give feedback to your tutor?	32	8.0	10	−2.918	0.516	0.004
Rate your overall level of satisfaction with the seminar?	32	9.0	9.0	−2.274	0.402	0.023
Rate your overall level of preference with the seminar?	32	5.0	9.0	−3.710	0.656	< 0.0001

## Discussion

With the sudden emergence of COVID-19 and mandated lockdowns, eLearning was the only alternative to ensure the continuation of the educational process [29]. This sudden shift was challenging for students, but more so for educators as there were several eLearning models to choose from without understanding which model would be more appropriate for dental students. VFL and VTL are among those eLearning models.

It is well established in the literature that flipped learning can help improve student engagement [30, 31]. This is because students get exposed to the course contents before the class, which allows for more interaction and engagement with their instructors and peers. In dentistry, flipped learning has shown successful outcomes compared to passive learning [32–34], This has also been the case for orthodontic courses [35]. However, VFL may require some adjustments that could affect the teaching outcomes. A recent paper compared VFL to traditional flipped learning and found no differences in course evaluations and the overall semester scores [16], encouraging educators to consider using VFL without any concerns regarding the effectiveness of this method. VFL can be implemented to deliver the didactic component of any preclinical or clinical course, especially since both dental students and educators have shown positive responses toward online learning when implemented during COVID-19 [36]. Given the versatility of eLearning modules available [37] and the positive perceptions by students [38], dental educators should utilize this to improve the dental curricula.

The current study showed that VTL and VFL had similar results in improving students’ short-term recall knowledge of the lectures’ content (Table 2). In other words, students demonstrated comparable short-term learning gains and knowledge retention regardless of the educational format. Similar findings were reported when comparing active and passive online learning modules using a crossover design [25], as well as when comparing the traditional flipped learning with the traditional teacher-lecture instructional method [39, 40]. Although this result may seem discouraging, it demonstrates that VFL did not result in an unfavorable effect on students’ knowledge retention as was demonstrated by written examination performance. It is possible, however, that other factors such as prior academic knowledge had an impact on the knowledge retention level as reported previously [41]. Moreover, it has been reported that novel tools such as clinical reasoning should be implemented to evaluate conceptual knowledge retention rather than traditional methods [42]. For this study, multiple choice questions were used as it is the form of assessment used mainly in this course and in the local licensing board exams.

Regarding learning effectiveness and outcomes, the current study showed no differences between the VFL and the VTL groups as measured by students’ performance on the post-test (positive-test raw scores) (Table 3). Similar results have been reported among medical students when passive online learning was compared to online active learning formats [25]. However, it should be noted that the small sample size of the study may have prevented detecting a difference between the two groups [43, 44]. This detection limit could also be the case for the current study and other similar studies. Previous studies have already established that flipped learning has more successful outcomes than passive learning [32–34, 45]. Again, it is possible that the format of the questions used in the present study in combination with the small sample size did not allow to capture this difference.

The students were more positive about their VFL format experience than the VTL format (Table 4). Significant differences were found among all the survey items, except for the opportunity to ask questions, suggesting that students had a better perception of VFL in general. Several studies have reported good satisfaction among students with the flipped learning method [46–48]. However, other studies have reported that students negatively viewed the flipped learning format, mostly in relation to the time of the online content [49–51]. It is possible that due to the nature of the current study where the student needs to sit in front of a computer regardless of the teaching method, students found more flexibility with the VFL. Regarding the opportunity to ask questions, it is possible that no differences were found as both learning formats allowed students to ask questions, either instantly during the VTL or later when the VFL was applied. The literature has some conflicting opinions regarding this matter. Some students reported that flipped learning allowed them to formulate better questions after watching the videos multiple times and became more confident and less afraid to participate during the class time [52, 53]. On the other hand, others preferred traditional learning as it provides an opportunity to ask questions in real-time and get instant feedback [54]. Therefore, it is possible to say that both formats give opportunities for the students to ask questions, however, it would be interesting in future studies to evaluate the quality of those questions asked by the students and see if differences exist between the two formats.

Given that the current study is among the first of its kind, especially in the context of dental education literature, future studies with larger samples are needed to investigate this issue. It is possible that different results could have been obtained if the sample size was larger. This study was conducted in a dental school with a limited number of registered students. However, the crossover design of this study allowed us to increase the statistical power of the study with fewer participants. Other design strengths of this study include having the opportunity to receive both interventions which can, on some occasions, be preferable by the subjects to participate in the study. The design, however, also has weaknesses such as carry-over effects of the intervention which can distort the recorded outcomes. Furthermore, completion of the study might take longer since the participants have to crossover into each arm in comparison to a parallel design [55–57]. Future studies should include several dental schools and conduct a parallel multi-center research study taking into account the standardization of lecture delivery. Another limitation in the present study was that only male students were included. The inclusion of female students was not possible as the trial was conducted at a gender-specific dental school. Future studies should include female students to evaluate if gender differences exist among dental students when comparing different asynchronous learning methods.

It is worth noting that there are some problems that can be associated with data collected from a questionnaire. For instance, response and non-response biases are two common issues that need to be considered when using such methods [58]. Response bias occurs when participants give systematically misleading answers. On the other hand, non-response bias refers to the low response rate which could result in an underrepresentation of the population being surveyed. Nonetheless, the questionnaire used in the current study is a validated Web-Based Learning Environment Inventory (WELEI) questionnaire [26].

## Conclusion

Contrary to the commonly perceived positive tangible impact of an in-person flipped classroom learning model, the current study demonstrates insignificant differences in learning gains within the VFL format. Such a finding poses the need to conduct further studies addressing the limitations of the current study, especially regarding different contexts, a larger sample size, long-term knowledge retention and attainment of higher cognitive skills. On the other hand, students preferred the virtual flipped learning format over the traditional virtual format. Therefore, dental educators are encouraged to carefully design online curricula with efficient learning strategies that help students improve learning performance and foster self-directed learning skills while valuing active learning in an online environment. To conclude, there is an important research scope to understand aspects related to barriers and facilitators of different formats of active online learning, including the virtual flipped learning format.

## Supplementary Information

**Additional file 1: Supplementary file 1.** Questionnaire for students

## Data Availability

The dataset that support the findings of this study are available on request from the corresponding author [FA]. The data are not publicly available due to containing information that could compromise research participant privacy/consent.

## Abbreviations

VTL: Virtual traditional learning method
VFL: Virtual flipped learning method
FL: Flipped learning
L1: First Lecture: Adult Orthodontics
L2: Second Lecture: Orthognathic Surgery
MCQs: Multiple-choice questions
WELEI: Web-Based Learning Environment Inventory
