# Supplementary material for: Comparison between two asynchronous teaching methods in an undergraduate dental course: a pilot study
Source: BMC Med Educ. 2022 Jun 23;22:488. doi: 10.1186/s12909-022-03557-7 (PMC9219382; doi:10.1186/s12909-022-03557-7)
Supplement: Supplementary file 1 — Additional file 1: Supplementary file 1. Questionnaire for students [file 12909_2022_3557_MOESM1_ESM.docx]

ID Group

Please circle a number on the scale from 0–10 where 0 **Not at all** and 10 **Very much**

1 Do you feel that you understood the topic that was being delivered today?

**0 1 2 3 4 5 6 7 8 9 10**

2 How interesting did you find the seminar?

**0 1 2 3 4 5 6 7 8 9 10**

3 Did you enjoy the seminar today?

**0 1 2 3 4 5 6 7 8 9 10**

4 Do you find it easy to concentrate?

**0 1 2 3 4 5 6 7 8 9 10**

5 Did you find it easy to participate in the session?

**0 1 2 3 4 5 6 7 8 9 10**

6 Was there an opportunity to ask questions?

**0 1 2 3 4 5 6 7 8 9 10**

7 Do you feel you were able to give feedback to your tutor?

**0 1 2 3 4 5 6 7 8 9 10**

8 Did you prepare for this seminar?

**0 1 2 3 4 5 6 7 8 9 10**

9 Do you feel you would perform better in exams for topics taught using flipped classroom ?

**0 1 2 3 4 5 6 7 8 9 10**

10 Do you prefer the conventional method of seminar teaching?

**0 1 2 3 4 5 6 7 8 9 10**

11 Do you prefer the flipped classroom method?

**0 1 2 3 4 5 6 7 8 9 10**

12 Will you be more likely to prepare for the next seminar if you know that flipped classroom will be used?

**0 1 2 3 4 5 6 7 8 9 10**

13 Overall, rate your level of satisfaction with the seminar?

0 1 2 3 4 5 6 7 8 9 10

Are there any comments you would like to make about lecture strategy using online and the flipped classroom? (Please continue overleaf if necessary) ----------------------------------------------------------------------- -------------------------------------------------------------------------------------------------------------------------------------------------------------------------------------------------------------------

--------------------------------------------------------------------------------------------------------------------------

ID Group

Please circle a number on the scale from 0–10 where 0 **Not at all** and 10 **Very much**

1 Do you feel that you understood the topic that was being delivered today?

**0 1 2 3 4 5 6 7 8 9 10**

2 How interesting did you find the seminar?

**0 1 2 3 4 5 6 7 8 9 10**

3 Did you enjoy the seminar today?

**0 1 2 3 4 5 6 7 8 9 10**

4 Do you find it easy to concentrate?

**0 1 2 3 4 5 6 7 8 9 10**

5 Did you find it easy to participate in the session?

**0 1 2 3 4 5 6 7 8 9 10**

6 Was there an opportunity to ask questions?

**0 1 2 3 4 5 6 7 8 9 10**

7 Do you feel you were able to give feedback to your tutor?

**0 1 2 3 4 5 6 7 8 9 10**

8 Did you prepare for this seminar?

**0 1 2 3 4 5 6 7 8 9 10**

9 Overall, rate your level of satisfaction with the seminar

**0 1 2 3 4 5 6 7 8 9 10**

Are there any comments you would like to make about lecture strategy using online and the flipped classroom? (Please continue overleaf if necessary) ----------------------------------------------------------------------- -------------------------------------------------------------------------------------------------------------------------------------------------------------------------------------------------------------------

--------------------------------------------------------------------------------------------------------------------------
